# Preventing mutant huntingtin proteolysis and intermittent fasting promote autophagy in models of Huntington disease

**DOI:** 10.1186/s40478-018-0518-0

**Published:** 2018-03-06

**Authors:** Dagmar E. Ehrnhoefer, Dale D. O. Martin, Mandi E. Schmidt, Xiaofan Qiu, Safia Ladha, Nicholas S. Caron, Niels H. Skotte, Yen T. N. Nguyen, Kuljeet Vaid, Amber L. Southwell, Sabine Engemann, Sonia Franciosi, Michael R. Hayden

**Affiliations:** 1 Centre for Molecular Medicine and Therapeutics (CMMT), CFRI, Department of Medical Genetics, University of British Columbia, 950 West 28th Avenue, Vancouver, BC V5Z 4H4 Canada; 2 Present address: BioMed X Innovation Center, Im Neuenheimer Feld 515, 69120 Heidelberg, Germany

**Keywords:** Huntington disease, Autophagy, Proteolysis, Caspase, Mutant huntingtin lowering

## Abstract

Huntington disease (HD) is caused by the expression of mutant huntingtin (mHTT) bearing a polyglutamine expansion. In HD, mHTT accumulation is accompanied by a dysfunction in basal autophagy, which manifests as specific defects in cargo loading during selective autophagy. Here we show that the expression of mHTT resistant to proteolysis at the caspase cleavage site D586 (C6R mHTT) increases autophagy, which may be due to its increased binding to the autophagy adapter p62. This is accompanied by faster degradation of C6R mHTT in vitro and a lack of mHTT accumulation the C6R mouse model with age. These findings may explain the previously observed neuroprotective properties of C6R mHTT. As the C6R mutation cannot be easily translated into a therapeutic approach, we show that a scheduled feeding paradigm is sufficient to lower mHTT levels in YAC128 mice expressing cleavable mHTT. This is consistent with a previous model, where the presence of cleavable mHTT impairs basal autophagy, while fasting-induced autophagy remains functional. In HD, mHTT clearance and autophagy may become increasingly impaired as a function of age and disease stage, because of gradually increased activity of mHTT-processing enzymes. Our findings imply that mHTT clearance could be enhanced by a regulated dietary schedule that promotes autophagy.

**Electronic supplementary material:**

The online version of this article (10.1186/s40478-018-0518-0) contains supplementary material, which is available to authorized users.

## Introduction

Huntington disease (HD) is an autosomal dominant neurodegenerative disorder that is caused by an expansion of a polyglutamine tract in the huntingtin (HTT) protein [57]. Mutant HTT (mHTT) causes dysfunction in different cellular compartments and pathways [13] that are difficult to target individually. The removal of mHTT itself is therefore an attractive therapeutic strategy and is currently being pursued in both clinical and pre-clinical studies [61]. While most of these studies aim to lower HTT RNA, changes in mHTT protein levels through increased degradation have also been shown to ameliorate HD symptoms [31, 59]. Both soluble and aggregated forms of mHTT are thought to be cleared preferentially through autophagy [47, 49], and both mTOR-dependent and -independent autophagic pathways have been implicated in its degradation [31, 48]. Interestingly, a role for HTT in the regulation of autophagy has recently been discovered [2, 34, 35, 43, 50]. Both the HTT N- and C-termini play different but inter-dependent roles in autophagy [43], which may be promoted by the interaction of the two halves after proteolysis [18]. However, multiple proteolytic events may disrupt the interaction between the HTT N- and C-termini [18]. The cleavage of mHTT in HD increases with disease progression and age, and may prevent HTT from functioning as an autophagy-promoting factor [21, 35].

Here we use the C6R mouse model, which expresses full-length mHTT with a mutation preventing proteolysis at amino acid 586 by caspases 6 and 8 [23, 62], to investigate the connection between mHTT cleavage and autophagy. We demonstrate a general increase in autophagy in cells and tissues from C6R mice compared to the YAC128 mouse model expressing fully cleavable full-length mHTT, and show that this is accompanied by reduced accumulation of mHTT protein.

HTT promotes autophagosome formation under basal, but not fasting conditions [50], suggesting that dietary interventions could circumvent mHTT-specific deficits in autophagy. In agreement with this hypothesis, we demonstrate that both fasting and scheduled feeding induce autophagy in the brains of HD mouse models, although only the longer scheduled feeding paradigm significantly reduces the levels of cleavable mHTT protein. These results provide a potential molecular mechanism for the beneficial effects of nutrient deprivation on neurodegenerative diseases [15, 31, 38, 54] and demonstrate that autophagy pathways that are not impacted by the HD pathology can be harnessed to lower mHTT protein levels in vivo.

## Results

### The expression of C6R mHTT promotes autophagy

HTT can act as a scaffold mediating cargo loading in basal autophagy [50], but as this function is dependent on the HTT C-terminus, it could be lost in HD due to proteolytic events [18, 35, 43]. To determine whether the expression of cleavable or C6R mHTT alters autophagy, we started by comparing mouse embryonic fibroblast (MEF) cultures derived from wt, YAC128 or C6R mice. The autophagy protein LC3-II decorates autophagosomes and is thus an indicator of autophagosome abundance, while the autophagy adapter protein p62 provides a link between LC3 and cargo proteins and is subsequently degraded together with the cargo [28]. Levels of these proteins are therefore commonly used to assess the autophagic state of cells [28].

**Fig. 1:**
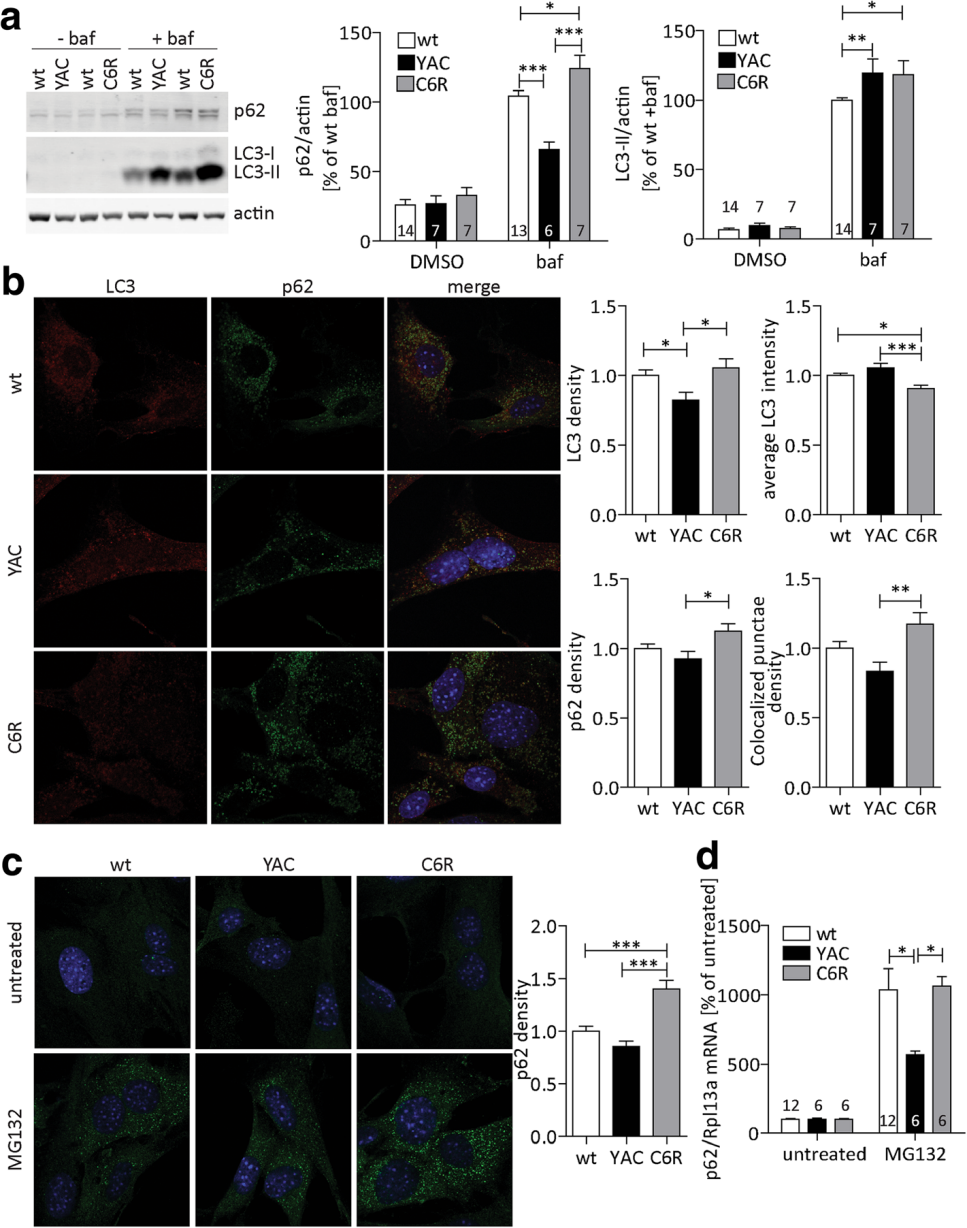
The expression of C6R mHTT promotes autophagy. **a** Primary MEF cultures from YAC128, C6R or wt littermate embryos were treated with bafilomycin or DMSO as a control. Levels of p62 and LC3-II were analyzed by Western blot. p62: 2way-ANOVA genotype *p* < 0.0001, bafilomycin *p* < 0.0001, LC3-II: 2way-ANOVA genotype *p* = 0.0239, bafilomycin *p* < 0.0001. **b** Primary MEF cultures from YAC128, C6R or wt littermate embryos were seeded onto coverslips and treated with bafilomycin. Cells were fixed and stained for p62 and LC3, Hoechst dye was used for nuclear counterstaining. Samples were imaged on a confocal microscope and the density of punctae, staining intensity as well as the co-localization of LC3 and p62 staining were analyzed. p62 density: 1way-ANOVA *p* = 0.0167, LC3 density: 1way-ANOVA *p* = 0.0116, LC3 staining intensity: 1way-ANOVA *p* = 0.0003, colocalization: 1way-ANOVA *p* = 0.0051. **c** Primary MEF cultures from YAC128, C6R or wt littermate embryos were seeded onto coverslips and treated with MG132 or DMSO as a control. Cells were fixed and stained for p62, Hoechst dye was used for nuclear counterstaining. Samples were imaged on a confocal microscope and the density of punctae was analyzed. 1way-ANOVA *p* < 0.0001. **d** Primary MEF cultures from YAC128, C6R or wt littermate embryos were seeded onto coverslips and treated with MG132 or DMSO as a control. RNA levels of p62 were determined by quantitative RT-PCR (qPCR), normalized to the expression level of Rpl13a. 2way-ANOVA: genotype *p* = 0.0461, treatment* p* < 0.0001. Representative blots/images and pooled quantification data with S.E.M. are shown, 3-5 independent cultures were analyzed. Number of replicates is shown as insets for Western blot and qPCR experiments, for imaging experiments 24-30 cells per condition were analyzed. Statistical significance was determined 2way-ANOVA with post-hoc Bonferroni correction. *: *p* < 0.05, **: *p* < 0.01, ***: *p* < 0.001

We found that baseline levels of p62 and LC3-II were similar between MEFs of all three genotypes, while the non-lipidated LC3-I was not detectable (Fig. 1a, higher intensity blots shown in Additional file 1: Figure S1A). Using bafilomycin, an inhibitor of autophagic flux, both p62 and LC3-II levels increased, as expected, since their turnover was blocked (Fig. 1a). However, the levels of p62 after bafilomycin treatment were lower in YAC128 MEFs compared to wt, while C6R MEFs showed no such deficit (Fig. 1a). At the same time, no reduction in LC3-II turnover was observed in bafilomycin-treated YAC128 MEFs by Western blotting (Fig. 1a). To quantify the formation of autophagosomes in more detail, we next analyzed the formation of punctae immunopositive for p62 and LC3 by confocal microscopy. While no such punctae were observed under basal conditions (Additional file 1: Figure S1B), treatment with bafilomycin led to an increase in both p62- and LC3-positive structures (Fig. 1b). Quantitative image analysis revealed that both the density of p62- and LC3-punctae was increased in cultures derived from C6R mice, whereas MEFs expressing cleavable YAC128 mHTT showed a trend towards a decrease compared to wt cultures in these measures (Fig. 1b), and were significantly different from C6R cells. Interestingly, C6R MEFs show an increased number of LC3 punctae density but lower LC3 signal intensity within those punctae (Fig. 1b), indicating that the available LC3 is distributed over a larger number of autophagosomes. Accordingly, also the density of punctae with colocalization of p62 and LC3 was increased in C6R MEFs, confirming their identity as autophagosomes (Fig. 1b).

In addition to its role as a cargo-binding protein in basal and starvation-induced autophagy, p62 also associates with misfolded proteins upon proteasomal inhibition and proteotoxic stress [14, 32]. In this context, we found that treatment with the proteasomal inhibitor MG132 led to a dramatic increase in p62-positive structures, which was again exacerbated in MEFs expressing C6R but not cleavable mHTT (Fig. 1c). Proteasomal inhibition leads to an accumulation of ubiquitinated proteins, which are then bound by p62 [29]. This can lead to a feedback loop of transcriptional upregulation of p62 through Nrf2 [14]. Indeed, we observed a strong upregulation of p62 mRNA expression after MG132 treatment (Fig. 1d), which was dampened in MEFs derived from YAC128 mice. Taken together, our data therefore suggest that cells derived from C6R mice upregulate autophagic pathways more efficiently than YAC128 cells, both at baseline and under conditions of proteotoxic stress, a situation most relevant to neurodegenerative diseases such as HD.

Interestingly, none of these measures were altered in MEFs derived from YAC18 mice overexpressing wild-type human HTT [24], suggesting that the changes in autophagosome formation are related to the C6R mutation in the mHTT protein, and not merely overexpression of a non-pathogenic variant of HTT (Additional file 2: Figure S2A + B).

### Reduced interaction between mHTT and p62 is normalized with the C6R mutation

**Fig. 2:**
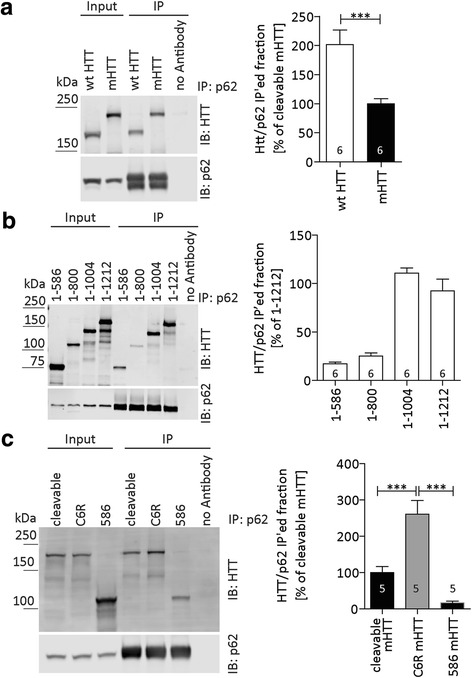
The C6R mutation improves the binding of mHTT to the autophagy adapter p62. **a** COS-7 cells were cotransfected with wt or mHTT aa1-1212 and p62 as indicated and treated with bafilomycin to block autophagic flux. After immunoprecipitation of p62, the ratio of co-immunoprecipitated HTT was quantified (normalized to input to control for transfection efficiency). **b** COS-7 cells were cotransfected with wt HTT fragments of different lengths and p62 as indicated and treated with bafilomycin to block autophagic flux. After immunoprecipitation of p62, the ratio of co-immunoprecipitated HTT was quantified (normalized to input to control for transfection efficiency). **c** COS-7 cells were cotransfected with cleavable mHTT_1-1212_, C6R mHTT_1-1212_ or mHTT_1-586_ and p62 as indicated and treated with bafilomycin to block autophagic flux. After immunoprecipitation of p62, the ratio of co-immunoprecipitated HTT was quantified (normalized to input to control for transfection efficiency). 1way-ANOVA *p* < 0.0001. Blots and quantification data with S.E.M. from a representative of 3 independent experiments are shown, number of technical replicates is shown as insets. Statistical significance was determined by Student’s t-test (**a**) or 1way-ANOVA with Tukey’s post-hoc correction (**c**). *: *p* < 0.05, **: *p* < 0.01, ***: *p* < 0.001

To further investigate the role of mHTT in autophagy, we next assessed its interaction with p62, and found that the presence of the expanded polyglutamine tract significantly reduced the binding efficiency of HTT_1-1212_ to p62 in co-transfected cells (Fig. 2a). As p62-interaction domains have only been described for the HTT C-terminus [43, 50] which is absent in our expression construct, we next decided to map this novel binding site. Using a series of truncation mutants, we determined that HTT interacts with p62 between amino acids 800-1004 (Fig. 2b), which is an area known to harbour an ULK1 binding domain and may therefore be involved in binding the ULK1/p62 complex during autophagosome formation [50].

Furthermore, we found that C6R mHTT_1-1212_ interacts approximately twice as strongly with p62 compared to cleavable mHTT (Fig. 2c), which was confirmed with co-immunoprecipitation of HTT with p62 (Additional file 3: Figure S3A). Consistent with our findings for wt HTT (Fig. 2b), the interaction between p62 and a mHTT_1-586_ fragment was also barely detectable, confirming that also for mHTT the p62 interaction domain is located C-terminal of the D586 caspase cleavage site (Fig. 2c). However, as the interaction is not completely abolished for the mHTT-586aa fragment, HTT may have additional p62 binding sites or multiple interaction domains that can be separated by proteolysis.

**Fig. 3:**
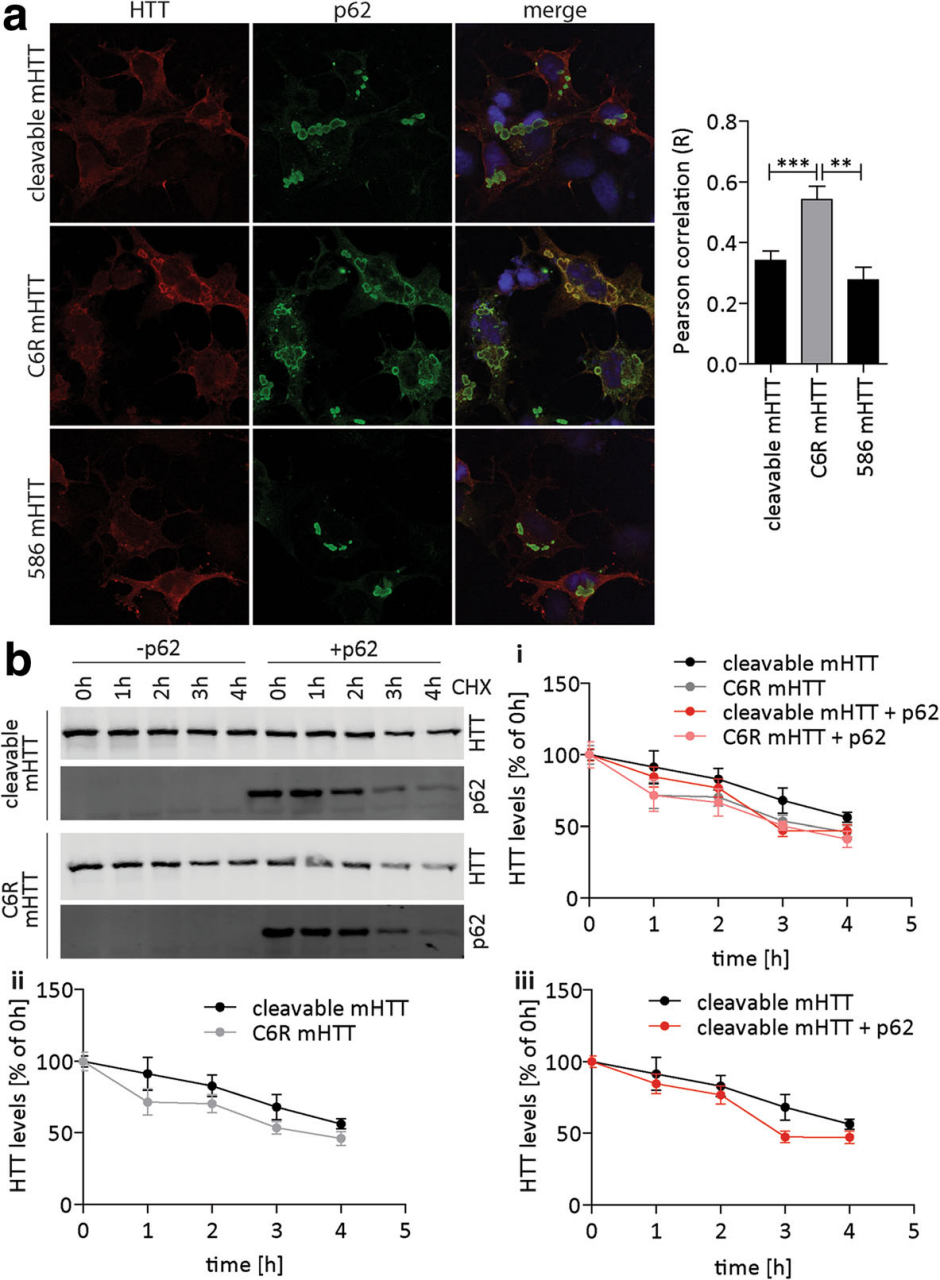
C6R mHTT preferentially colocalizes with p62 and is degraded faster than cleavable mHTT. **a** COS-7 cells were seeded onto coverslips, cotransfected with cleavable mHTT_1-1212_, C6R mHTT_1-1212_ or mHTT_1-586_ and p62 as indicated and treated with bafilomycin to block autophagic flux. Cells were fixed and stained for p62 and HTT, Hoechst dye was used for nuclear counterstaining. Samples were imaged on a confocal microscope and the colocalization of HTT and p62 signals was analyzed. 1way-ANOVA *p* < 0.0001. **b** COS-7 cells were cotransfected with cleavable mHTT_1-1212_, C6R mHTT_1-1212_ and p62 as indicated and treated with MG132 to enforce autophagic degradation. Cycloheximide was added for the indicated periods of time and samples were analyzed by Western blot. Data are graphed in three different versions to better visualize differences between groups: (i) all four conditions, (ii) cleavable mHTT vs. C6R mHTT, 2way-ANOVA mHTT construct *p* = 0.0146, time *p* < 0.0001, (iii) cleavable mHTT without/with p62, 2way-ANOVA p62 *p* = 0.0436, time *p* < 0.0001. Representative blots/images and pooled quantification data with S.E.M. are shown, for imaging experiments 14-21 cells per condition were analyzed, for Western blotting 3 independent experiments were performed with 3 technical replicates each. Statistical significance was determined by 1way-ANOVA with Tukey’s post-hoc correction for A and 2way-ANOVA with Bonferroni’s post-hoc correction for B. *: *p* < 0.05, **: *p* < 0.01, ***: *p* < 0.001

Immunofluorescence staining confirmed the preferential localization of C6R mHTT to p62-positive areas (Fig. 3a), which was much reduced for cleavable mHTT or the mHTT-586aa fragment. This colocalization may relate to the role of HTT in autophagosome formation [43, 50], but may also influence the autophagic clearance of HTT itself. We therefore performed cycloheximide-chase experiments in COS cells co-transfected with cleavable or C6R mHTT_1-1212_ and p62. In the absence of p62 overexpression, cleavable mHTT is degraded more slowly than C6R mHTT (Fig. 3b) within the first 4 h of chase time. However, when p62 is overexpressed, the degradation of cleavable mHTT is accelerated and rendered indistinguishable from C6R mHTT (Fig. 3b), suggesting that a sufficiently large supply of p62 can overcome degradation deficits of cleavable mHTT. Overexpressed p62 was almost completely degraded within the 4 h timeframe of the experiment (Fig. 3b, quantified in Additional file 3: Figure S3B). We furthermore confirmed that the decrease in full-length HTT and p62 levels are not due to proteolysis, since no accumulation of degradation products was observed on the full Western blots (shown in Additional file 4: Figure S4C).

p62 interacts with ubiquitinated substrates, and preferentially those that are linked to ubiquitin through lysine 63 (K63), promoting their autophagic degradation [4, 53]. HTT can be ubiquitinated by K63 or K48 linkages, and both types of ubiquitinated mHTT accumulate in cell and mouse models of HD, which has been attributed to impaired clearance by both autophagy and the proteasome [6, 7]. Co-transfection of cleavable or C6R mHTT_1-122_ with either wt ubiquitin, or ubiquitin mutants that can only bind their target proteins through lysine 48 (K48 ubiquitin) or lysine 63 (K63 ubiquitin), revealed that C6R mHTT co-immunoprecipitated with significantly more ubiquitin in general (wt ubiquitin, Additional file 3: Figure S3C). Interestingly, the interaction with K48 ubiquitin was equal between cleavable and C6R mHTT, but K63 ubiquitin preferentially co-immunoprecipitated with C6R mHTT, indicating that the K63 linkage is preferred in the presence of the C6R mutation (Additional file 3: Figure S3C). Increased K63-ubiquitination of C6R mHTT would thus be expected to mediate increased p62 binding and may therefore account for its preferential autophagic clearance.

### Fasting-induced autophagy is functional in the presence of mHTT

As a next step, we decided to investigate autophagy pathways in vivo. Since the liver heavily relies on autophagy to maintain its basal function [33], and HD-specific dysfunction in autophagic and metabolic pathways has been found in livers from HD mouse models and human patients [9, 36, 58, 59], we decided to focus on both brain and liver tissues from YAC128 and C6R mice.

**Fig. 4:**
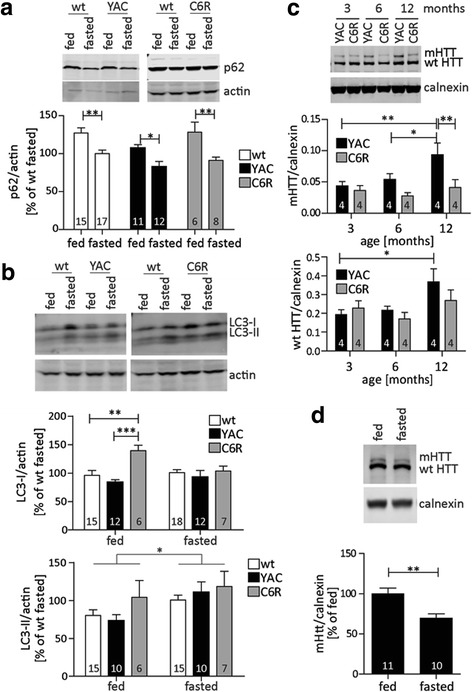
mHTT levels increase in aging YAC128 liver and can be reduced by fasting-induced autophagy. **a**, **b** + **d** 12 month old YAC128 and C6R mice, as well as their wt littermates, were subjected to a 24 h fasting period, sacrificed immediately and liver samples were compared to littermates with ad libitum access to food. p62 (**a**) and LC3 (**b**) protein levels were analyzed by Western blot. p62: 2way-ANOVA genotype *p* = 0.0103, fasting *p* < 0.0001, LC3-I: 2way-ANOVA genotype *p* = 0.0043, fasting *p* = 0.3161, LC3-II: 2way-ANOVA genotype *p* = 0.2012, fasting *p* = 0.0151. **c** Liver tissues from YAC128 and C6R mice at different ages were analyzed for HTT expression using the MAB2166 antibody. mHTT 2way-ANOVA genotype *p* = 0.0047, age *p* = 0.0342; wt HTT 2way-ANOVA genotype *p* = 0.3168, age *p* = 0.0232. **d** HTT protein levels in YAC128 liver were analysed by Western blot using the MAB2166 antibody. Representative blots and pooled quantification data with S.E.M. are shown, number of replicates is shown as insets. Statistical significance was determined by 2way-ANOVA with Bonferroni’s post-hoc correction for **a** - **c**, or two-tailed Student’s t-test for **d**. *: *p* < 0.05, **: *p* < 0.01, ***: *p* < 0.001

We first compared baseline levels of autophagy with a food deprivation paradigm, which is expected to activate autophagy [12]. A fasting period of 24 h was sufficient to observe significant changes in hepatic levels of key autophagy proteins in wt, YAC128 and C6R mice: fasting decreased p62 levels, in agreement with its increased autophagic turnover following food deprivation (Fig. 4a) [28]. Furthermore, LC3-II levels were increased by fasting (Fig. 4b), indicating enhanced autophagosome formation. Interestingly, LC3-I levels were strikingly elevated in C6R mice under fed conditions (Fig. 4b). Fasting eliminated this increase (Fig. 4b), suggesting that fasting leads to a rapid conversion of available LC3-I pools into LC3-II. This was further analyzed by qRT-PCR, which showed similar expression levels of LC3 for mice of all three genotypes at baseline (Additional file 5: Figure S5A), demonstrating that the differences observed by Western blotting are post-transcriptional.

To determine whether alterations in autophagy had an impact on the degradation of mHTT, we next assessed HTT protein levels in the liver of YAC128 and C6R mice. We found a strong age-dependent increase in wt and mHTT protein that reached statistical significance at 12 months in YAC128 animals (Fig. 4c). On the other hand, C6R mice showed no age-dependent alterations in wt or mHTT levels, suggesting that this change is specific to the expression of cleavable mHTT (Fig. 4c). To confirm that the changes are post-transcriptional, we performed qRT-PCR analyses on liver tissues from 12 month old mice. Interestingly, mHTT mRNA levels are higher in C6R compared to YAC128 liver tissues (Additional file 5: Figure S5B), confirming that the lack of mHTT accumulation observed by Western blot are not due to decreased expression, but rather due to post-transcriptional effects such as increased protein degradation.

Fasting-induced autophagy in the liver was paralleled by a significant reduction of mHTT protein in YAC128 mice (Fig. 4d), while the levels of wt HTT remained unchanged (Additional file 5: Figure S5C). mRNA levels of the mHTT transgene were also not affected by fasting, confirming that this intervention likely reduced mHTT protein through autophagic degradation (Additional file 5: Figure S5D). Fasting also had an impact on hepatic mHTT protein levels in C6R mice, although the reduction was more subtle in this genotype (Additional file 5: Figure S5E). This is not surprising, given the already low levels of mHTT protein in C6R compared to YAC128 mice. Nevertheless, the trend towards a further decrease suggests that fasting-induced autophagy can still lower mHTT even in C6R mice.

Taken together, these findings demonstrate that basal autophagy is altered in the liver of C6R mice and may be responsible for the lack of age-dependent mHTT accumulation in this mouse model. Fasting-induced autophagy mechanisms on the other hand are intact and can be activated in the liver of both YAC128 and C6R mice. Furthermore, our data suggest that the age-dependent accumulation of mHTT can be reversed by activating such protein degradation pathways simply through dietary changes.

### Prolonged regulation of food intake induces mHTT clearance in the brain

Recent studies have demonstrated that acute fasting also induces autophagy in the CNS [1, 10]. We therefore examined LC3 and p62 levels in cortical tissues from fasted as well as mice fed ad libitum, and found that a 24 h fasting period increased LC3-I, LC3-II and p62 protein levels in the cortex of both wt and YAC128 animals (Additional file 6: Figure S6A). While both the increased cortical p62 and LC3-I levels differ from our findings in the liver (Fig. 4a and b), this may suggest different timing of autophagy induction in the two organs. These differences not only manifest on the protein level, but are also observed transcriptionally: While YAC128 mice show a trend towards reduced p62 expression in both the liver and the cortex at baseline (Additional file 6: Figure S6B + C), fasting induces a reduction in p62 mRNA in the liver but not the cortex of wt animals (Additional file 6: Figure S6B + C). Furthermore, unlike hepatic mHTT (Fig. 4d), cortical mHTT levels remained unaffected by a 24 h fasting period (Additional file 6: Figure S6D). We therefore hypothesized that the lack of mHTT degradation after acute fasting may be due to the delayed induction of autophagy in the brain compared to the liver, and designed a fasting schedule that could be maintained for a longer period of time.

**Fig. 5:**
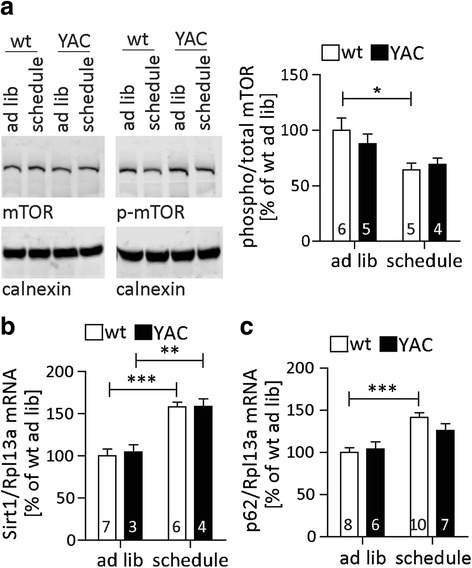
Scheduled feeding alters nutrient-sensing pathways in the brain. **a - c** YAC128 mice and their wt littermates were subjected to 1 week of scheduled feeding and compared to littermates with ad libitum access to food. **a** Levels of total and phosphorylated mTOR were analyzed by Western blot. 2way-ANOVA genotype *p* = 0.6796, feeding *p* = 0.0082. **b + c** mRNA levels of SIRT1 (**b**) and p62 (**c**) were analyzed by qRT-PCR. SIRT1: 2way-ANOVA genotype *p* = 0.7651, feeding *p* < 0.0001, p62: 2way-ANOVA genotype *p* = 0.4121, feeding *p* < 0.0001. Representative blots and pooled quantification data with S.E.M. are shown. Statistical significance was determined by 2way-ANOVA with Bonferroni’s post-hoc correction, number of replicates is shown as insets. *: *p* < 0.05, **: *p* < 0.01, ***: *p* < 0.001

In our paradigm, mice were fasted for 18 h and allowed full access to food for 6 h periods. We hypothesized that this schedule would induce autophagy in vivo daily, without necessarily reducing caloric intake. Long-term caloric restriction has beneficial effects on aging and neurodegenerative phenotypes [11, 42], and previous studies have implicated both mTOR inhibition and SIRT1 activation in these phenomena [11, 20, 25, 26]. Consistent with these reports, we observed that our scheduled feeding diet decreased phosphorylated mTOR (Fig. 5a), an indication of mTOR inhibition similar to the effect of nutrient deprivation [64]. In addition, RNA levels of SIRT1 were increased in mice subjected to scheduled feeding (Fig. 5b), which can further contribute to the downregulation of mTOR signaling [20] and thus induce autophagy.

**Fig. 6:**
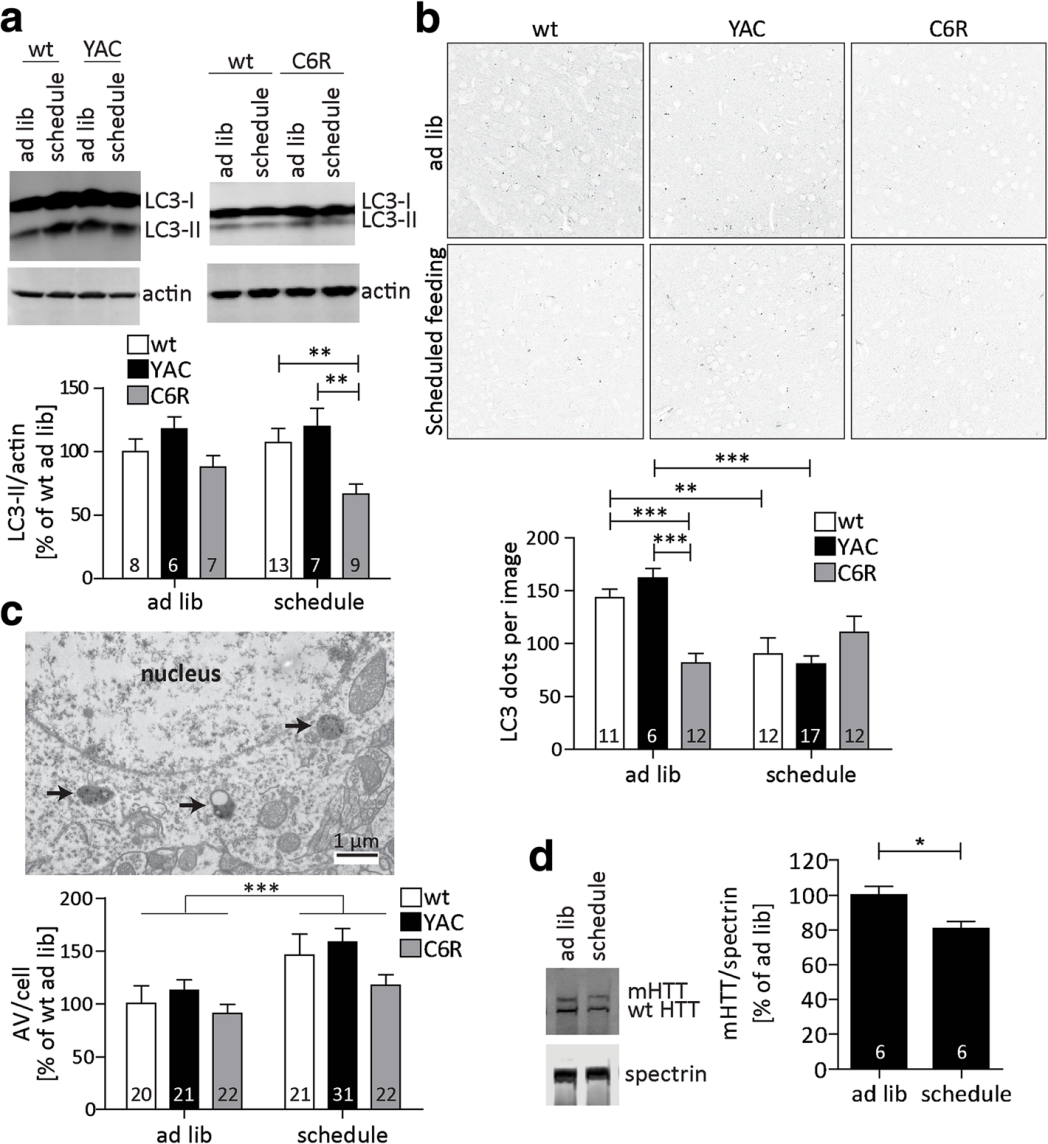
Scheduled feeding induces autophagy and lowers mHTT protein in the brain. **a - d** YAC128 and C6R mice as well as their wt littermates were subjected to 1 week of scheduled feeding and cortical tissue was compared to littermates with ad libitum access to food. **a** LC3-II levels in cortical lysates were determined by Western blotting. 2way-ANOVA genotype *p* = 0.0034, feeding *p* = 0.6776. **b** Cortical brain sections were stained for LC3 and confocal images were analyzed. 2way-ANOVA genotype *p* = 0.0815, feeding *p* = 0.0006. **c** Brain sections of the motor cortex were analyzed by EM, and autophagic vesicles (AV) surrounding the nuclei were counted in a blinded fashion. 2way-ANOVA genotype *p* = 0.0636, feeding *p* = 0.0006. **d** mHTT protein levels in cortical tissues were analyzed by Western blotting using antibody MAB2166. Representative images/blots and pooled quantification data with S.E.M. are shown, number of replicates is shown as insets. Statistical significance in **a** - **c** was determined by 2way-ANOVA with Bonferroni’s post-hoc correction, in D by two-tailed Student’s t-test. *: *p* < 0.05, **: *p* < 0.01, ***: *p* < 0.001

While the steady-state cortical p62 protein levels were not altered by scheduled feeding (Additional file 7: Figure S7A), we observed a significant induction of p62 expression at the RNA level (Fig. 5c). This is consistent with the previously demonstrated replenishing of p62 protein levels through increased expression during long-term fasting [5, 51]. Although LC3-I levels were not altered by scheduled feeding (Additional file 7: Figure S7B), C6R mice responded to the paradigm with significantly lower levels of LC3-II compared to wt or YAC128 animals (Fig. 6a). Furthermore, confocal microscopy demonstrated a strong reduction in LC3 punctae in C6R mice at baseline, with levels that are only reached after scheduled feeding in wt and YAC128 mice (Fig. 6b). Taken together, these data point towards higher autophagic flux in the CNS of C6R mice, but also suggest that it is possible to reach similar autophagy levels in YAC128 animals through scheduled feeding.

Next, EM analysis was performed to quantify the formation of autophagosomes and autophagolysosomes in the cortex of scheduled-fed mice. In this experiment we observed predominantly autolysosomes with electron-dense content with some remaining ultrastructure [28], which are the most abundant form of autophagic vesicles (AVs) in neurons [8]. We found that the overall number of AVs per cell significantly increased after scheduled feeding, confirming that autophagosomes are successfully transported to the neuronal soma and cargo degradation increased (Fig. 6c). Consistent with this data we found that the scheduled feeding paradigm also significantly reduced the levels of cortical mHTT protein in YAC128 mice (Fig. 6d), similar to the effect of short-term fasting on mHTT the liver (Fig. 4d). No changes were observed for wt HTT protein (Additional file 7: Figure S7C) or mHTT mRNA (Additional file 7: Figure S7D). Taken together our data therefore suggests that scheduled feeding increases mHTT clearance in the brain through the upregulation of autophagy, and that this mechanism is functional in a mouse model expressing cleavable mHTT.

## Discussion

The expansion of the CAG tract in HTT is the single cause for HD. Recent efforts in therapeutic development have therefore focused on different strategies to lower the levels of mHTT [61]. While a major focus of these efforts lies on the reduction of mHTT expression, there is strong evidence for dysfunction of mHTT clearance pathways in HD [30]. In particular, impaired autophagy has been linked to the well-documented accumulation of mHTT in the CNS [30, 39].

Here, we show that the expression of mHTT resistant to proteolytic cleavage at D586 (C6R mHTT) leads to increased basal and proteotoxicity-induced autophagy in primary MEFs. Together with the finding that autophagy is normal in MEFs derived from mice overexpressing wt HTT (YAC18), our data therefore suggest that the C6R mutation in particular causes the observed alterations in autophagy pathways. This may be due to an altered structure of C6R compared to cleavable mHTT, since we also find that C6R mHTT preferentially interacts with p62 compared to the full-length form of cleavable mHTT. As this interaction site localizes to an area also bound by ULK1 [50], the aa800-1004 region of HTT may form an ULK1/p62/HTT complex that can initiate autophagosome formation [34, 35, 50]. At the same time, the increased interaction may promote the autophagic degradation of C6R mHTT itself. This mechanism may explain why C6R mice fail to accumulate mHTT in the liver with age, despite sufficient expression levels and in contrast to YAC128 animals. C6R mice are thus better protected from the accumulation of toxic, aggregated forms of mHTT compared to YAC128 animals, which may shed light on the remarkable lack of HD phenotypes in the former mouse model [21, 23, 40, 45].

**Fig. 7:**
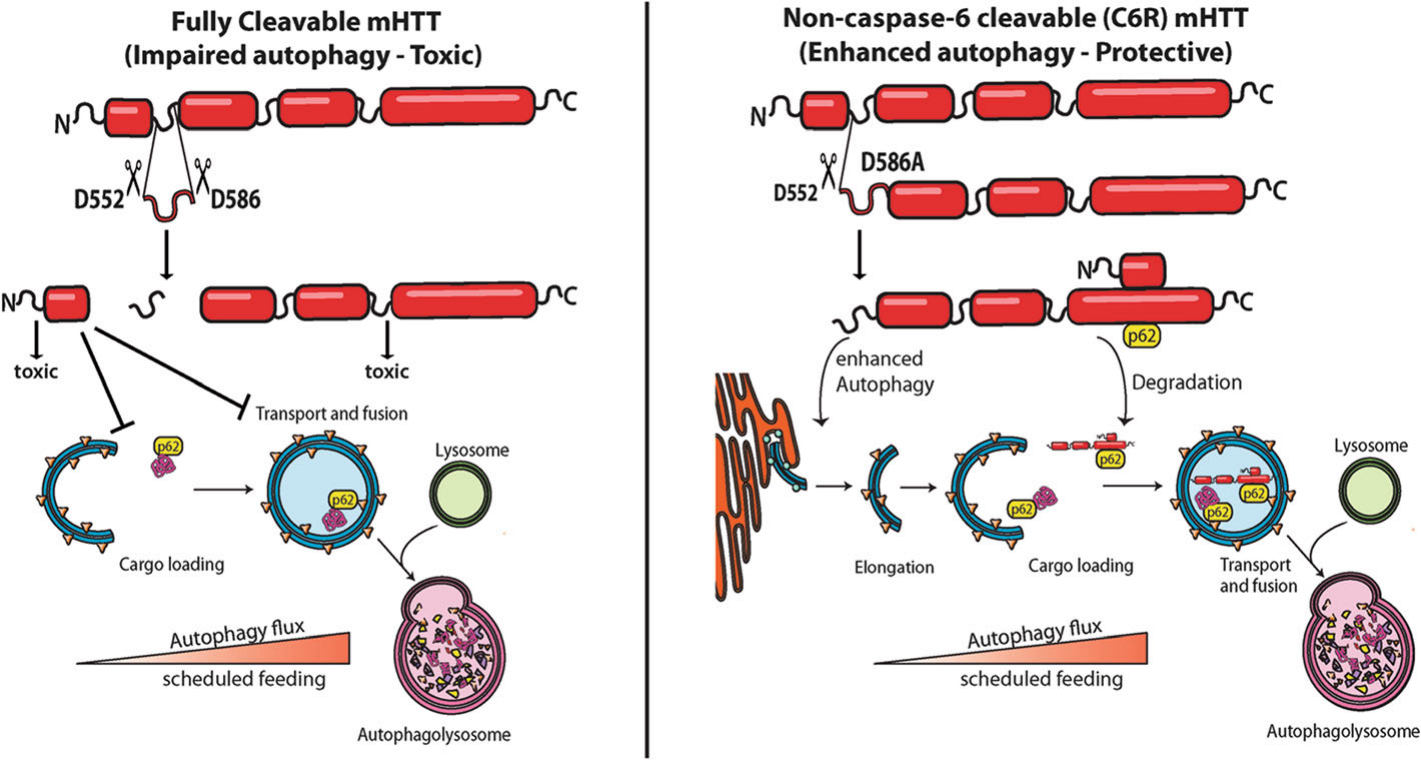
Schematic representation of the effects of cleavable or C6R mHTT on autophagy. mHTT is subject to proteolysis by different proteases, with a number of cleavage sites clustering in the PEST2 domain [17]. Cleavage at D552 and D586 liberate a small fragment that is myristoylated at G553 [34] and induces autophagosome formation. However, both the N-terminal and C-terminal fragments resulting from mHTT double cleavage are toxic and interfere with autophagic cargo loading and autophagosome transport and fusion [18, 36, 43, 63]. In the C6R mice, cleavage at D586 is prevented, and we observed enhanced autophagy as well as improved degradation of C6R mHTT. In both YAC128 and C6R mice, autophagic flux and mHTT degradation can be enhanced in peripheral tissues by fasting and in the CNS by scheduled feeding

We only observe very subtle and specific deficits in autophagy in MEFs derived from YAC128 mice: Western blotting revealed a decrease in p62 turnover, with no deficit in LC3, while defects in autophagosome formation monitored by immunofluorescence were also less pronounced. However, the cargo recognition failure and specific defects in selective, but not bulk autophagy reported previously for HD model systems [36, 50] may be related to the decreased ability of mHTT to bind p62. This defect may not only prevent the degradation of mHTT, but through impaired formation of p62/ULK1/HTT complexes could impact selective autophagy in general. Recent studies have shown that the C-terminus of the HTT protein can act as a scaffold similar to Atg11, and promote autophagosome formation [43]. This function is regulated by an interaction between the HTT N- and C-termini [43], which is disrupted in the case of multiple proteolytic events within the HTT protein [18]. We therefore propose a model in which the negative effects of mHTT N-terminal fragments on autophagosome formation, transport and fusion are repaired in C6R mice since mHTT cleavage at D586 is prevented (Fig. 7). This furthermore boosts the normal function of the intact HTT C-terminus in promoting autophagosome formation and more efficient degradation of mHTT itself (Fig. 7). Supporting this hypothesis, increased autophagy and reduced mHTT levels have been demonstrated previously in HD mouse models crossed to a C6^−/−^ line, an intervention that reduces (but does not completely abolish) mHTT cleavage at D586 [19, 62].

Although the C6R mutation is beneficial, it is not directly translatable to human HD patients. We therefore set out to test a therapeutic intervention that has the potential to alter autophagy pathways in vivo and to monitor its effects in the presence of cleavable mHTT. Caloric restriction can slow aging in a large variety of animal models [37] and upregulate key transcription factors such as *SIRT1* that are beneficial in different neurodegenerative conditions including HD [11, 25, 26]. However, such an intervention is not advisable for human patients, since HD already leads to a significant reduction in body weight [3] which may be exacerbated by further caloric reduction. We show here that scheduled feeding is sufficient to upregulate *SIRT1* expression and activate the mTOR pathway in a mouse model of HD. Importantly, intermittent fasting can still trigger starvation-induced autophagy and mHTT clearance in the YAC128 mouse model of HD, even though the overall calorie intake was not restricted. Furthermore, subtle deficits in autophagic pathways caused by the expression of cleavable mHTT did not prevent autophagy induction, suggesting that any such defects can be overcome by strong autophagy-inducing stimuli.

Circadian rhythms are disrupted in HD patients as well as in animal models of the disease, and this phenotype can be ameliorated by forcing a circadian pattern of food intake in mice, even at late stages of the disease [38]. Since autophagy follows a circadian pattern in the brain [1], it is possible that the disruption of circadian rhythms in neurodegenerative disease may cause autophagic dysfunction and contribute to the accumulation of autophagy substrates such as mHTT. Furthermore, treating disruptions in circadian rhythm through lifestyle changes may ameliorate symptoms such as depression, anxiety and cognitive dysfunction in human HD patients [41], and our data suggest that such an intervention has the potential to lower mHTT protein levels through increased autophagy.

## Conclusions

In this study, we provide evidence that not only prolonged fasting but also scheduled feeding without forcibly reducing calorie intake alters nutrient-sensing pathways and activates autophagy in mouse brain. This intervention furthermore reduces the amounts of mHTT protein, and may thus contribute to its clearance. As mHTT levels are closely correlated with pathology, these findings therefore correlate environmental influences with disease in a mouse model of HD.

In addition, we show that dysregulation of autophagy caused by the expression of mHTT is not observed when the protein is rendered resistant to cleavage at D586 (C6R mHTT). Age-dependent accumulation of mHTT is curtailed in C6R mice, and increased autophagy observed in cells derived from this mouse model may be responsible for the puzzling lack of HD phenotypes in these animals [21, 23, 40, 45].

## Materials and methods

### Animal models and statistics

All mouse experiments were carried out in accordance with protocols (Animal protocol A07-0106) approved by the UBC Committee on Animal Care and the Canadian Council on Animal Care. Mice are derived from in-house breeding pairs, maintained under a 12 h light:12 h dark cycle in a clean facility and given free access to food and water except otherwise indicated (for fasting and scheduled feeding protocols). YAC128 (line HD53 [56]) and C6R (line W13 [23]) mice are on a FVB/N background, mixed sexes were analyzed. Cortex and liver tissue was dissected and snap-frozen on dry ice for protein analyses.

Sample sizes were chosen based on extensive experience with biochemical differences between YAC128 mice and their WT littermates for experiments using mouse tissues [21–23, 44, 46, 58, 62]. Cell culture experiments were repeated independently at least three times to ensure reproducibility. Samples were only excluded if technical issues were apparent (i.e. bubble on a Western blot) or if determined statistical outliers using Grubb’s outlier test (α = 0.05, no more than one sample per group was excluded).

For randomization, mice were assigned numbers not related to genotype. Scientists performing experiments were blinded for genotypes, unless it was necessary to ensure the appropriate order of samples on a gel. Data analyses was performed by a separate person in possession of the genotype information. For image analysis of electron microscopy and confocal microscopy data, unblinding was performed after all quantitation was complete.

Statistical significance was assessed using Student’s t-test for comparison of two groups, one-way ANOVA with post-hoc Tukey’s correction for the comparison of one variable between more than two groups, and two-way ANOVA with post-hoc Bonferroni correction for the comparison of two variables between groups. Variances between groups were similar. All analyses were performed using the GraphPad Prism 5.01 software package.

### Generation of primary cell cultures

Primary MEF and neuronal cultures from YAC128 (line 53, [56]), YAC18 (line 212 [24]) and C6R (line W13, [23]) embryos, as well as their wt littermates were set up as described previously [16, 55]. In brief, embryos were collected on day 15.5–16.5 of gestation for neuronal cultures and at day 12.5 for MEF cultures. Tissues were extracted and transferred to Hibernate E (Invitrogen) for up to 24 h, during which time samples from the remaining embryonic tissues were genotyped [55]. For MEFs, the body without head, limbs, liver, lung and heart was minced, digested with 0.25% trypsin-EDTA and taken up in MEF medium (Dulbeccos’s modified Eagle medium with high glucose, 10% fetal calf serum, 2 mM L-Glutamine, 100 μM non-essential amino acids, 1 mM sodium pyruvate, 1 μ M β-mercaptoethanol). Cells were triturated with a pipette tip, digested with DNAse I and a single cell suspension without clumps was seeded. When indicated, cells were treated with 20 nM bafilomycin A1 (Cayman Chemicals) or DMSO for 16 h. Cells were harvested by scraping and lysed for Western blot analysis as described [16] or fixed for immunofluorescence.

For neuronal cultures, cortices were micro-dissected in ice-cold Hank’s balanced salt solution (HBSS+; Gibco), then diced and pooled for each genotype. Cells were dissociated with 0.05% trypsin-EDTA (Gibco), followed by neutralization with 10% fetal calf serum in neuro basal medium (NBM+) and DNAse I treatment (153 U/mL). Tissue was triturated with a pipette five to six times. Cells were plated on poly-D-lysine coated 6-well plates with 2 ml of Neurobasal media (Gibco #21103-049), B27 (Gibco #17504-044), 100 U/mLpenicillin-streptomycin (PS) (Gibco), 0.5 mM L-glutamine and maintained at 37°C, 5% CO2 with humidity. Cells were fed with 200 mL fresh medium every fifth day. On day 9-11 in culture, cells were treated with 10 nM bafilomycin A1 (Cayman Chemicals) or DMSO for 2 h. Cells were harvested by scraping and lysed for Western blot analysis as described [16].

### Western blotting

Western blots were performed on samples lysed in SDP lysis buffer (50 mM Tris pH 8, 150 mM NaCl, 1% Igepal with ‘Complete’ protease inhibitor cocktail (Roche)). Protein concentration was measured using the DC protein assay kit (Bio Rad, USA) and equal amounts were separated on 7% Bis-Tris gels for the detection of HTT, or 4-12% gradient gels (Invitrogen, USA) for the detection of LC3 and p62. Protein was transferred to PVDF Immobilon-FL membranes by electroblotting and membranes were developed with primary antibodies in 5% bovine serum albumin/phosphate buffered saline. The following antibodies were used for immunoblotting: anti-HTT BKP1 (1:100) generated in-house [27], anti-HTT 2166 (1:1000) from Millipore (MAB2166), anti-polyglutamine expansion antibody (1C2, MAB1574) from Millipore (1:2000), anti-p62 (1:1000) from ENZO (BML-PW9860), anti-LC3b (1:1000) from Cell Signaling Technologies (2775), anti-mTOR (1:1000) from Cell Signaling Technologies (2983), anti-phospho-mTOR (1:1000) from Cell Signaling Technologies (5536), anti-HA (1:1000) from COVANCE (MMS-101R), anti-Actin (1:10,000) from Sigma (A2103), anti-calnexin (1:5000) from Sigma (C4731), anti-spectrin (1:4000) from ENZO (bm-FG6090). Fluorescently labelled secondary antibodies conjugated to either 700 or 800 IR dye (1:5000; Rockland, USA) and the LiCor Odyssey Infrared Imaging system were used for detection. The following antibodies were used for immunocytochemistry experiments: anti-p62 from R&D Systems (MAB8028; 1:200), anti-LC3β from Cell Signaling (2775S; 1:200), anti-polyglutamine expansion antibody (1C2, MAB1574;1:1000) from Millipore, Alexa Fluor 488 goat anti-mouse IgG from Invitrogen (A11001; 1:500), and Alexa Fluor 568 goat anti-rabbit IgG from Invitrogen (A11011; 1:500).

### Immunocytochemistry

Cells (MEFs or COS-7) were cultured on coverslips in 24-well plates and treated with bafilomycin (16 h; 20 nM), MG132 (4 h; 10 μM), or DMSO followed by fixation with 4% paraformaldehyde in PBS for 15 min at room temperature (RT). Cells were treated with ice-cold methanol for 5 min at − 20 °C, washed 3× in PBS, permeabilized in 0.03% Triton-X/PBS for 5 min at RT, washed 3× in PBS, and incubated in blocking buffer (0.2% gelatin/PBS) at RT for 30 min. Coverslips were transferred to primary antibody solution made up in blocking buffer and incubated overnight at 4 °C, followed by 3xPBS washes and incubation with secondary antibody in blocking buffer at RT for 1.5 h. Coverslips were washed and mounted on slides with ProLong Gold antifade reagent with DAPI (Molecular Probes).

### Confocal imaging and image analysis

Single z-plane images were acquired on a Leica TCS SP8 confocal laser scanning microscope at 63X objective magnification. Images were imported into Image J, background subtracted using a rolling ball radius of 15 pixels, and de-speckled. 1-3 cells per image were analysed by selecting 3 random regions of interest (ROIs) within the cytosol of each cell, manually thresholding punctae from each channel, and evaluating punctae size, density, and overlap using the Image J Colocalization plugin and the Analyse Particles function. All data were normalized to the mean of the appropriate wild-type littermate control values.

For COS cell analysis, punctae analysis was not possible due to the diffuse staining pattern of transfected HTT. Instead, 1-3 cells per image were outlined to generate ROIs, and the Coloc2 plugin was utilized to calculate a Pearson correlation coefficient as measures of colocalization between channels.

### COS-7 cell transfection and immunoprecipitation

COS-7 cells were maintained in DMEM with 10% FBS, 1% L-glutamine, 100 U/mL penicillin and 0.1 mg/mL streptomycin at 37 °C and 5% CO2 in a humidified incubator. HTT expression constructs aa1-1212 and 1-586 were described previously [60], fragments aa1-800 and aa1-1004 were generated from HTT_1-1212_ 15Q using the Q5 site-directed mutagenesis kit (NEB) with the following primers:

- HTT 800 For: 5′ - AACCCTCACATGAAATACATTTTCTTTG - 3’
- HTT 800 Rev.: 5′ - CTAATGGTGCCCATCCAATC - 3’
- HTT 1004 For: 5′ - AAATAACCTTTGAAGAGTTATTGCAG - 3’
- HTT 1004 Rev.: 5′ - TCCATAGTGACGTCTGTTATG - 3’

Correct insertion of the stop codons was verified by sequencing.

Cells were transiently cotransfected with the aa1-1212 15Q, aa1-586 15Q, aa1-800 15Q, aa1-1004 15Q, aa1-1212 128Q (cleavable), aa1-1212 138Q-C6R or aa1-586 Q128 HTT constructs (11) together with RFP-p62 (obtained from Addgene (12)) or HA-ubiquitin wt, K63 or K48 (obtained from Addgene (13)). The Xtreme gene 9 transfection reagent (Roche Applied Science, Quebec, Canada) was used according to the manufacturer’s protocol.

The day after transfection, cells were treated with 100 nM bafilomycin for 4 h to prevent HTT-p62 and HTT-ubiquitin complexes from degradation. Cell lysates were prepared in SDP buffer and immunoprecipitated over night at 4 °C using anti-p62 (MBL PM045) or anti-HTT 2166 antibodies (Millipore MAB2166). Immunoprecipitates and cell lysates were subjected to SDS-PAGE and Western blot as described above.

### Cycloheximide chase assay

COS-7 cells were transfected as above. 6 h after transfection, 10 μM MG132 were added to prevent proteasomal degradation and enforce autophagic clearance of mHTT. 16 h later, 0 h timepoints were harvested and 100 μg/ml cycloheximide were added for the indicated timepoints. Samples were lysed and analyzed by Western blotting as described above.

### qRT-PCR

RNA was extracted using the PureLink mini RNA extraction kit (Life Technologies). RNA was treated with DNase I (Invitrogen) and 500 ng of RNA were reverse transcribed using SuperScript III (Invitrogen) and oligo-dT primers according to manufacturer’s instructions to generate cDNA for qRT-PCR. The PCR was run with SYBR Green Power master mix (Applied Biosystems) on the ABI Prism 7500 Sequence Detection System.

Each sample was run in triplicate. Relative gene expression was determined by using the ΔΔC_T_ method, normalizing to *Rpl13a* mRNA levels in cortical tissues and MEF cells and to *Pgk1* mRNA levels in liver. The following primers were used:

- Human *Htt* forward: 5′- GAAAGTCAGTCCGGGTAGAAC -3’
- Human *Htt* reverse: 5′- CAGATACCCGCTCCATAGCAA -3′
- mouse *Rpl13a* forward: 5’-GGAGGAGAAACGGAAGGAAAAG-3′
- mouse *Rpl13a* reverse: 5′- CCGTAACCTCAAGATCTGCTTCTT-3′
- mouse *Pgk1* forward: 5′ - ACCTGCTGGCTGGATGG - 3′
- mouse *Pgk1* reverse: 5′ - CACAGCCTCGGCATATTTCT - 3′
- mouse *Sirt1* forward: 5′- CAGTGTCATGGTTCCTTTGC -3′
- mouse *Sirt1* reverse: 5′– CACCGAGGAACTACCTGAT -3′
- mouse *p62* forward: 5′- CTCAGCCCTCTAGGCATTG – 3′
- mouse *p62* reverse: 5′- TCCTTCCTGTGAGGGGTCT – 3′
- mouse *LC3b1* forward: 5′ - CTCACTCGTGGTCTGAGGACTTC - 3′
- mouse *LC3b1* reverse: 5′ - GGTGGCTATGCTGGCTTCA - 3′

### Transmission electron microscopy (TEM)

Mice were anesthetized with avertin and injected with 15 μL of heparin intracardially. Mice were perfused with 4% paraformaldehyde and 0.125% glutaraldehyde for 20 min at a rate of 6 mL/min. Brains were dissected and left overnight in fixative at room temperature. 400 μm sections were cut on a vibratome and 1 mm^2^ tissue blocks of motor cortex were dissected. Postfixing, embedding, sectioning and staining were performed at the University of British Columbia BioImaging facility. Briefly, samples were rinsed in 0.1 M sodium cacodylate buffer and secondary fixed in 1% osmium tetroxide with 1.5% potassium ferricyanide in 0.1 M sodium cacodylate for 2 h. Tissue was washed two times in distilled water and dehydrated in a series of ethanol dilutions, followed by graded resin infiltration and embedding. Ultrathin sections were prepared on a Leica Ultracut 7 using 45 degree diamond histoknife. Thin sections were counterstained with Sato’s lead. Images were taken using a Hitachi H7600 Transmission Electron Microscope and analyzed using Image J. 10-15 cells per mouse were identified and pictures taken systematically around the nucleus, covering all visible cytoplasm. 4-5 mice per genotype from 2 to 4 separate litters of YAC and wt mice were analyzed in a randomized and blinded fashion. AV were identified as either autophagosomes with a double membrane and visible cytoplasmic content such as mitochondria, or autolysosomes (vesicles with electron-dense content and some remaining ultrastructure) [28] and counted manually. The number of AV/cell was calculated and normalized to wt littermates. Imaging and counting were performed by separate blinded investigators.

### Immunohistochemistry and image analysis

Samples from the same mouse brains as used in electron microscopy were mounted on slides and stained with anti-LC3b (1:1000) from Cell Signaling Technologies (2775). Slides were imaged on a Leica SP5 laser-scanning confocal microscope with a 63X immersion plan-apochromat objective. Fixed and stained mouse cortices were imaged at 100 Hz with a 1024 × 1024 pixel scan format, a zoom factor of 1 and a pinhole size of 75 μm. LC3 staining was imaged using the 543 laser at 15% laser power (50% intensity and 100% gain) and DAPI was imaged using the UV laser at full laser power (25% intensity and 10% gain).

Background subtraction on the LC3 image stack was performed using the rolling ball method using a radius of 10 pixels. A threshold mask was then applied to the image stack based on intensities ranging from 100 to 255 to generate binary images. Subsequently, ImageJ [52] automated particle analysis was performed on the image stack and particle counts, size and area were measured for all images.

## Additional files

Additional file 1: **Figure S1.** LC3 and p62 are barely detectable at baseline in MEF cultures. **A** Higher exposure of blots shown in Fig. 1a demonstrates low baseline levels of p62 and LC3 in MEF cells. **B** Immunofluorescent staining for LC3 and p62 is barely detectable in MEFs in the absence of bafilomycin. (TIFF 2834 kb)

Additional file 2: **Figure S2.** Autophagy pathways are not altered in MEFs derived from YAC18 mice. **A** Primary MEF cultures from YAC18 or wt littermate embryos were seeded onto coverslips and treated with bafilomycin. Cells were fixed and stained for p62 and LC3, Hoechst dye was used for nuclear counterstaining. Samples were imaged on a confocal microscope and the density of punctae as well as the co-localization of LC3 and p62 staining were analyzed. **B** Primary MEF cultures from YAC18 or wt littermate embryos were seeded onto coverslips and treated with MG132 or DMSO as a control. Cells were fixed and stained for p62, Hoechst dye was used for nuclear counterstaining. Samples were imaged on a confocal microscope and the density of punctae were analyzed. Representative images and pooled quantification data with S.E.M. are shown, 3 independent cultures were analyzed. Number of replicates is shown as insets for Western blot experiments, for imaging experiments 24-30 cells per condition were analyzed. Statistical significance was determined by Student’s t-test. No statistically significant differences were found. (TIFF 5239 kb)

Additional file 3: **Figure S3.** Increased association of p62 and K63 ubiquitin with C6R mHTT. **A** COS-7 cells were cotransfected with mHTT aa 1-1212 (cleavable or C6R) or mHTT aa 1-586 and p62 as indicated. After immunoprecipitation of HTT, the ratio of co-immunoprecipitated p62 was quantified (normalized to input to control for transfection efficiency). **B** COS-7 cells were cotransfected with cleavable mHTT_1-1212_, C6R mHTT_1-1212_ and p62 as indicated and treated with MG132 to enforce autophagic degradation. Cycloheximide was added for the indicated periods of time and samples were analyzed by Western blot. Representative blots are shown as part of Fig. 3b. 2way-ANOVA HTT construct p=0.1451, time *p*<0.0001. **C** COS-7 cells were cotransfected with mHTT aa1-1212 (cleavable or C6R) and HA-tagged wt, K63 or K48 ubiquitin (allowing all, only K63 or only K48 linkage to target proteins) as indicated. After immunoprecipitation of HTT, the ratio of co-immunoprecipitated ubiquitin/HTT was quantified (normalized to input to control for transfection efficiency). Blots and quantification data with S.E.M. from a representative of 3 independent experiments are shown, number of technical replicates is shown as insets. Statistical significance was determined by 1way ANOVA with Tukey’s post-hoc correction (A), 2way-ANOVA with Bonferroni’s post-hoc correction (B) or Student’s t-test (D). *: *p*<0.05, **: *p*<0.01, ***: *p*<0.001. (TIFF 2089 kb)

Additional file 4: **Figure S4.** Full blots corresponding to Fig. 3b. (TIFF 4590 kb)

Additional file 5: **Figure S5.** 24h fasting does not alter wt HTT protein or mHTT RNA levels while it reduces C6R mHTT in the liver. **A** Liver tissue from 12 month old YAC128 and C6R mice as well as their wt littermates was analyzed for mRNA expression of *LC3b* by qRT-PCR. Data were normalized to the expression of *Pgk1*. 1way-ANOVA *p*=0.6552. **B** Liver tissue from 12 month old YAC128 and C6R mice was analyzed for mRNA expression of *mHTT* by qRT-PCR. Data were normalized to the expression of *Pgk1*. **C - E** 12 month old YAC128 and C6R mice as well as their wt littermates were subjected to a 24h fasting period, sacrificed immediately and liver samples were compared to littermates with ad libitum access to food. **C** Protein levels for wt HTT were analyzed by Western blotting with antibody MAB2166 in liver tissues derived from YAC128 mice. **D** mRNA levels for transgenic human mHTT were analyzed by qRT-PCR in liver tissues derived from YAC128 mice. **E** Protein levels for mHTT were analyzed by Western blotting with antibody MAB2166 in liver tissues derived from C6R mice. Representative blots and pooled quantification data with S.E.M. are shown, the blot matching panel C is shown in Fig. 5d. Statistical significance was determined by 1way-ANOVA (A) or two-tailed Student’s t-test (B-E), number of replicates is shown as insets. (TIFF 1130 kb)

Additional file 6: **Figure S6.** 24h fasting increases autophagy but does not cause mHTT degradation in the brain. **A + B** 3 month old YAC128 and C6R mice as well as their wt littermates were subjected to a 24h fasting period, sacrificed immediately and cortical samples were compared to littermates with ad libitum access to food. **A** Protein levels of p62, LC3-I and LC3-II were analyzed by Western blotting in cortical tissues of wt and YAC128 mice. p62: 2way-ANOVA genotype *p*=0.2568, feeding *p*=0.0002, LC3-I: 2way-ANOVA genotype *p*=0.7914, feeding *p*<0.0001, LC3-II: 2way-ANOVA genotype *p*=0.5499, feeding *p*=0.0039. **B** Protein levels of mHTT were analyzed by Western blotting with antibody MAB2166 in cortical tissues of YAC128 mice. Representative blots and pooled quantification data with S.E.M. are shown, number of replicates is shown as insets. Statistical significance was determined by 2way-ANOVA with Bonferroni’s post-hoc correction for A, or two-tailed Student’s t-test for B. *: *p*<0.05, **: *p*<0.01, ***: *p*<0.001. (TIFF 1574 kb)

Additional file 7: **Figure S7.** Cortical p62, LC3-I and wt HTT protein levels as well as mHTT mRNA are not altered by scheduled feeding. **A** YAC128 and C6R mice as well as their wt littermates were subjected to one week of scheduled feeding and compared to littermates with ad libitum access to food. Protein levels of p62 were analyzed by Western blotting in cortical tissues. 2way-ANOVA, genotype *p*=0.2138, feeding *p*=0.5807. **B** YAC128 and C6R mice as well as their wt littermates were subjected to one week of scheduled feeding and compared to littermates with ad libitum access to food. Protein levels of LC3-I were analyzed by Western blotting in cortical tissues. 2way-ANOVA, genotype *p*=0.5798, feeding *p*=0.2548. **C + D** YAC128 mice and their wt littermates were subjected to one week of scheduled feeding and compared to littermates with ad libitum access to food. **C** Protein levels of wt HTT were analyzed by Western blotting with antibody MAB2166 in cortical tissues. 2way-ANOVA genotype *p*=0.6115, feeding *p*=0.1818. **D** mRNA levels for transgenic human mHTT were analyzed by qRT-PCR in cortical tissues derived from YAC128 mice. Representative blots and pooled quantification data with S.E.M. are shown, number of replicates is shown as insets. The blot corresponding to panel B is shown in Fig. 6a, the blot corresponding to panel C is shown in Fig. 6d. Statistical significance was determined by 2way ANOVA (A-C) or Student’s t-test (D). (TIFF 1304 kb)
