# Supplementary figures and images for: Preventing mutant huntingtin proteolysis and intermittent fasting promote autophagy in models of Huntington disease
Source: Acta Neuropathol Commun. 2018 Mar 6;6:16. doi: 10.1186/s40478-018-0518-0 (PMC5839066; doi:10.1186/s40478-018-0518-0)

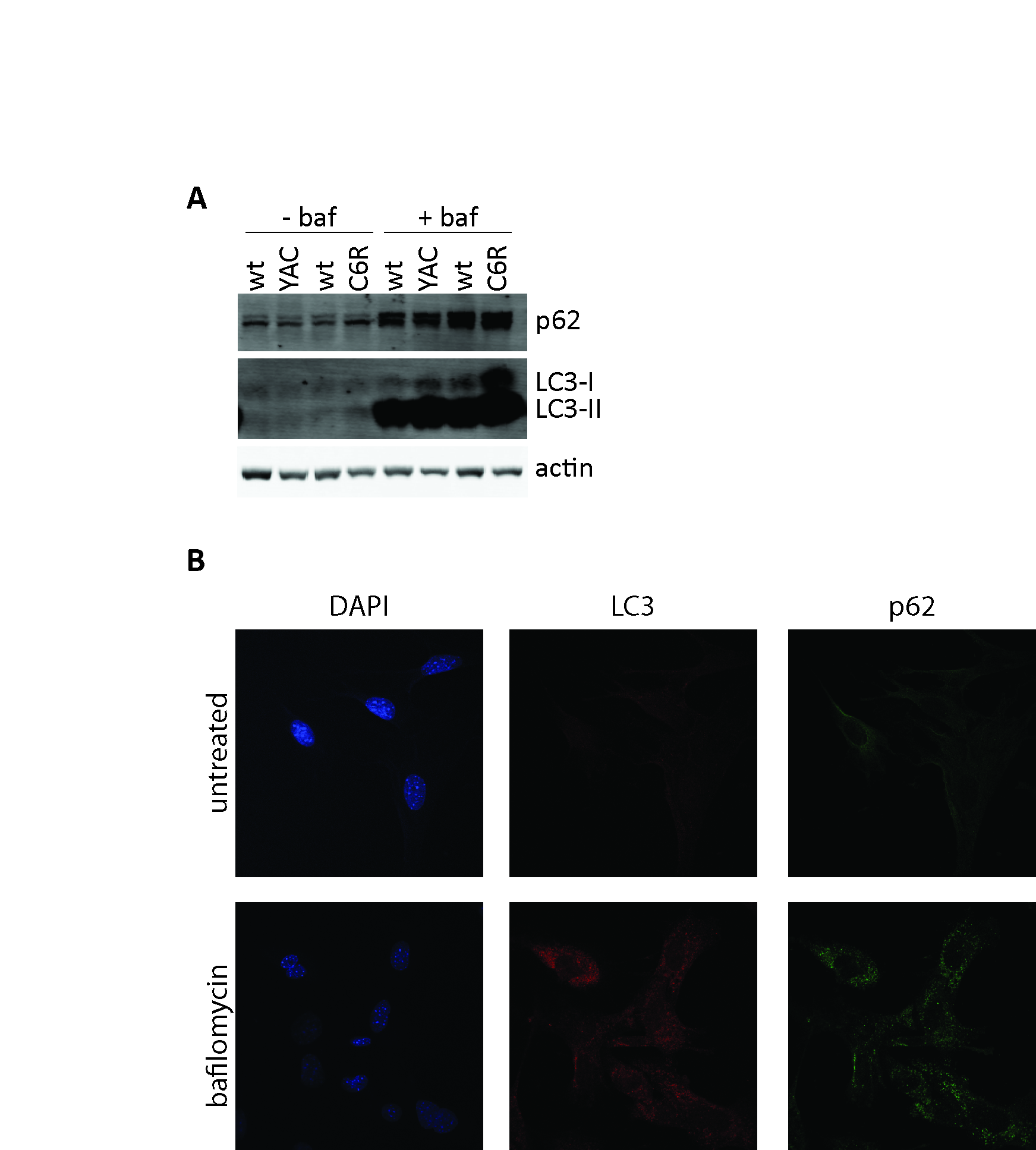

Supplement: Supplementary file 1 — Figure S1. LC3 and p62 are barely detectable at baseline in MEF cultures. A Higher exposure of blots shown in Fig. 1a demonstrates low baseline levels of p62 and LC3 in MEF cells. B Immunofluorescent staining for LC3 and p62 is barely detectable in MEFs in the absence of bafilomycin. (TIFF 2834 kb) [file 40478_2018_518_MOESM1_ESM.tif]

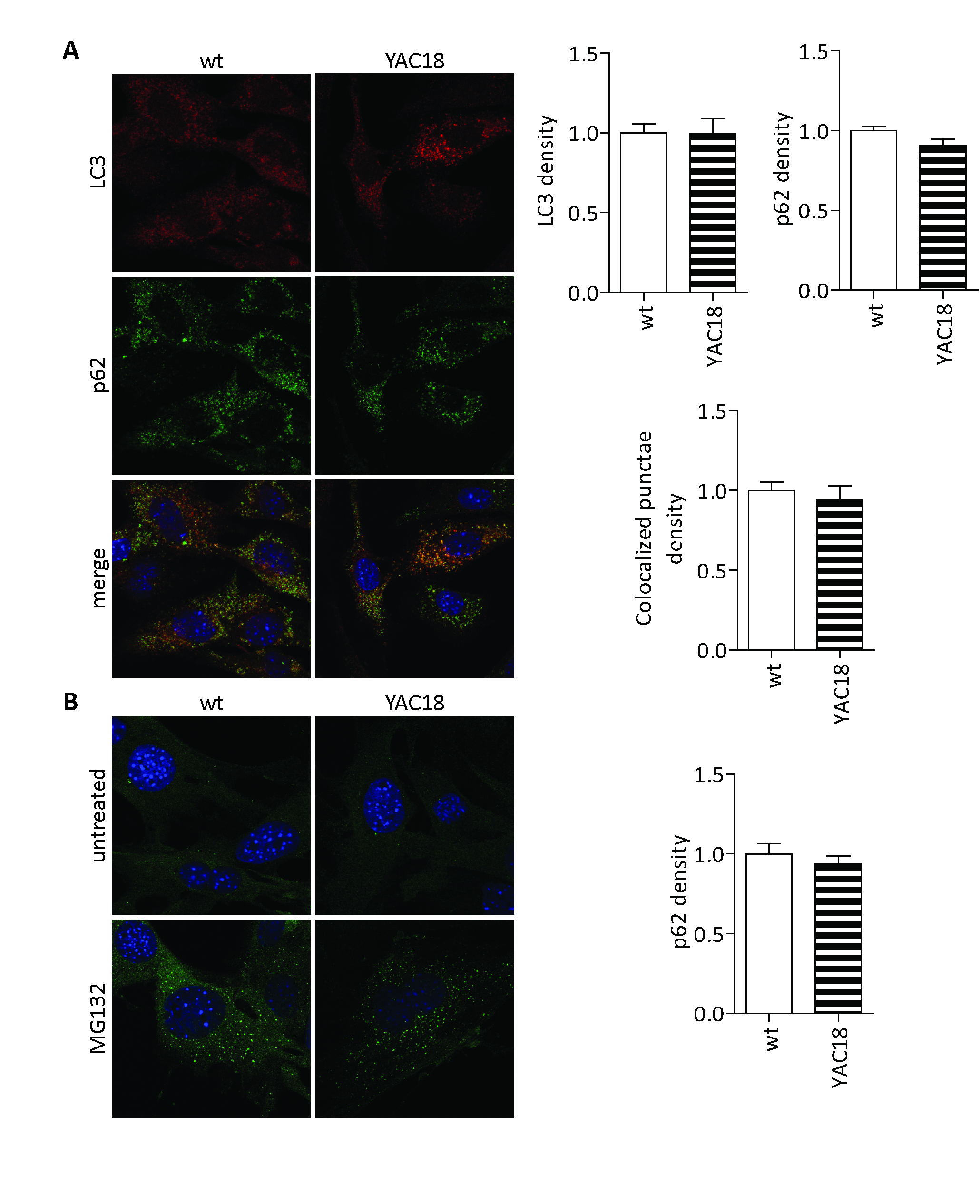

Supplement: Supplementary file 2 — Figure S2. Autophagy pathways are not altered in MEFs derived from YAC18 mice. A Primary MEF cultures from YAC18 or wt littermate embryos were seeded onto coverslips and treated with bafilomycin. Cells were fixed and stained for p62 and LC3, Hoechst dye was used for nuclear counterstaining. Samples were imaged on a confocal microscope and the density of punctae as well as the co-localization of LC3 and p62 staining were analyzed. B Primary MEF cultures from YAC18 or wt littermate embryos were seeded onto coverslips and treated with MG132 or DMSO as a control. Cells were fixed and stained for p62, Hoechst dye was used for nuclear counterstaining. Samples were imaged on a confocal microscope and the density of punctae were analyzed. Representative images and pooled quantification data with S.E.M. are shown, 3 independent cultures were analyzed. Number of replicates is shown as insets for Western blot experiments, for imaging experiments 24-30 cells per condition were analyzed. Statistical significance was determined by Student’s t-test. No statistically significant differences were found. (TIFF 5239 kb) [file 40478_2018_518_MOESM2_ESM.tif]

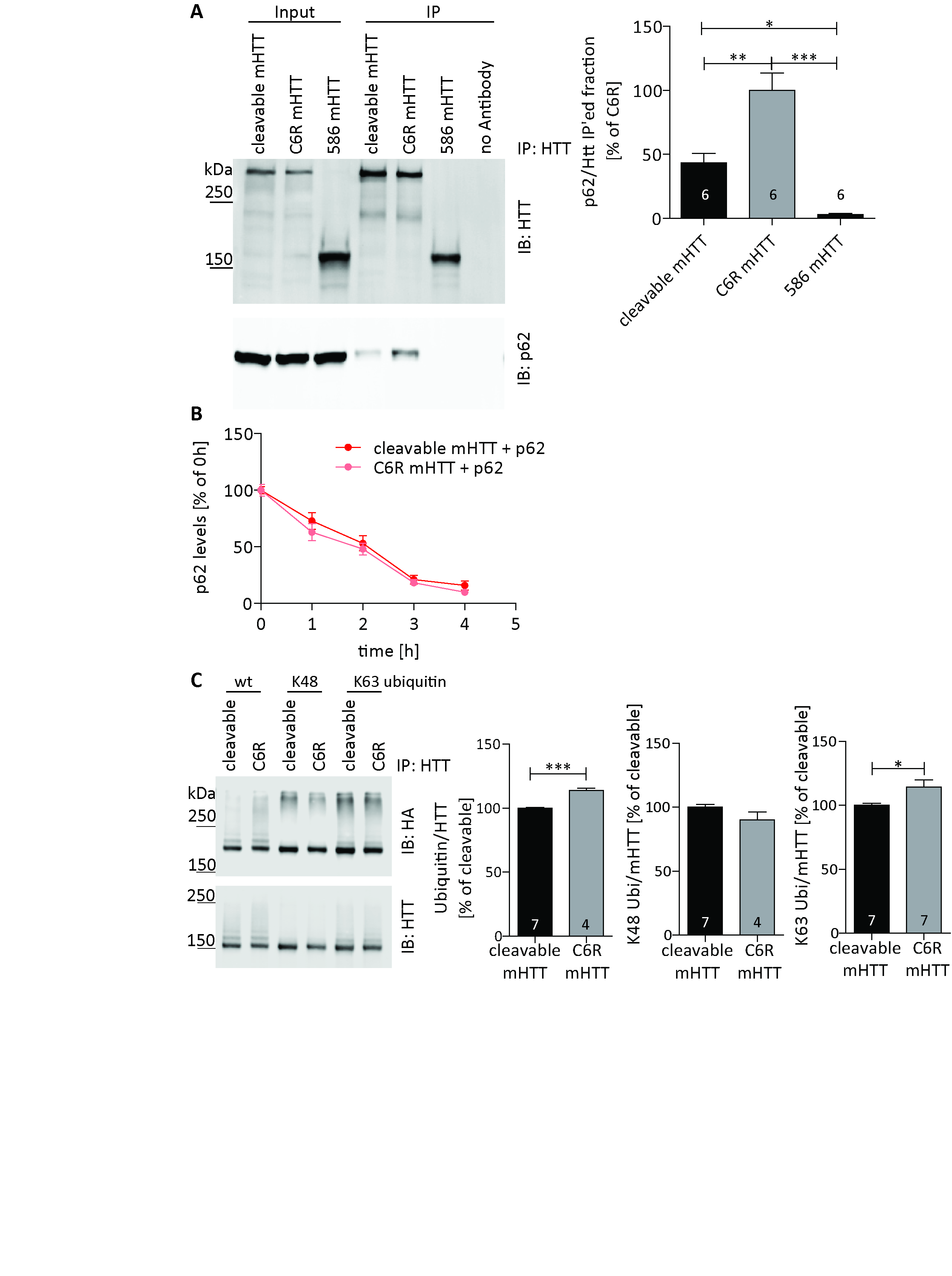

Supplement: Supplementary file 3 — Figure S3. Increased association of p62 and K63 ubiquitin with C6R mHTT. A COS-7 cells were cotransfected with mHTT aa 1-1212 (cleavable or C6R) or mHTT aa 1-586 and p62 as indicated. After immunoprecipitation of HTT, the ratio of co-immunoprecipitated p62 was quantified (normalized to input to control for transfection efficiency). B COS-7 cells were cotransfected with cleavable mHTT1-1212, C6R mHTT1-1212 and p62 as indicated and treated with MG132 to enforce autophagic degradation. Cycloheximide was added for the indicated periods of time and samples were analyzed by Western blot. Representative blots are shown as part of Fig. 3b. 2way-ANOVA HTT construct p=0.1451, time p<0.0001. C COS-7 cells were cotransfected with mHTT aa1-1212 (cleavable or C6R) and HA-tagged wt, K63 or K48 ubiquitin (allowing all, only K63 or only K48 linkage to target proteins) as indicated. After immunoprecipitation of HTT, the ratio of co-immunoprecipitated ubiquitin/HTT was quantified (normalized to input to control for transfection efficiency). Blots and quantification data with S.E.M. from a representative of 3 independent experiments are shown, number of technical replicates is shown as insets. Statistical significance was determined by 1way ANOVA with Tukey’s post-hoc correction (A), 2way-ANOVA with Bonferroni’s post-hoc correction (B) or Student’s t-test (D). *: p<0.05, **: p<0.01, ***: p<0.001. (TIFF 2089 kb) [file 40478_2018_518_MOESM3_ESM.tif]

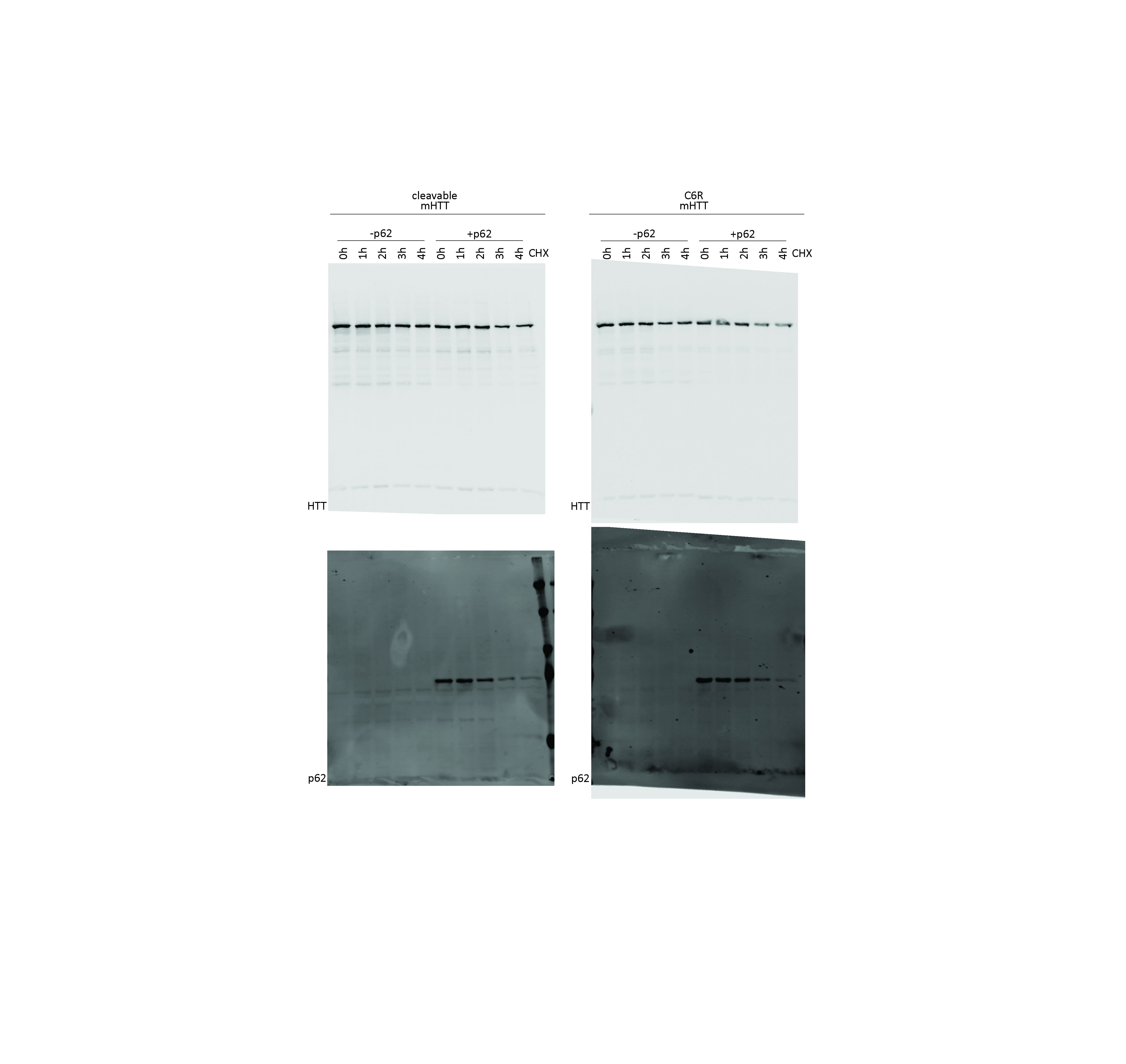

Supplement: Supplementary file 4 — Figure S4. Full blots corresponding to Fig. 3b. (TIFF 4590 kb) [file 40478_2018_518_MOESM4_ESM.tif]

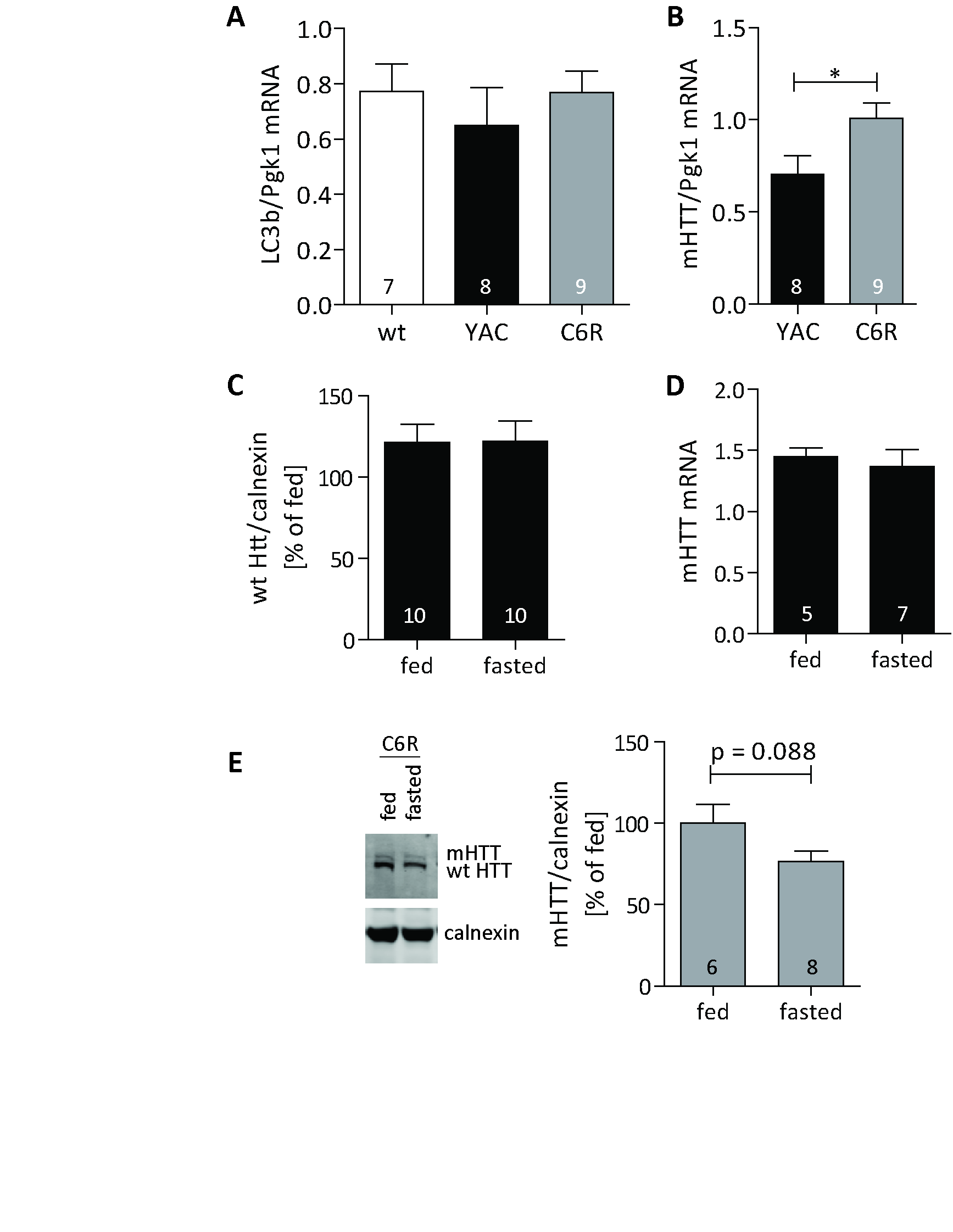

Supplement: Supplementary file 5 — Figure S5. 24h fasting does not alter wt HTT protein or mHTT RNA levels while it reduces C6R mHTT in the liver. A Liver tissue from 12 month old YAC128 and C6R mice as well as their wt littermates was analyzed for mRNA expression of LC3b by qRT-PCR. Data were normalized to the expression of Pgk1. 1way-ANOVA p=0.6552. B Liver tissue from 12 month old YAC128 and C6R mice was analyzed for mRNA expression of mHTT by qRT-PCR. Data were normalized to the expression of Pgk1. C - E 12 month old YAC128 and C6R mice as well as their wt littermates were subjected to a 24h fasting period, sacrificed immediately and liver samples were compared to littermates with ad libitum access to food. C Protein levels for wt HTT were analyzed by Western blotting with antibody MAB2166 in liver tissues derived from YAC128 mice. D mRNA levels for transgenic human mHTT were analyzed by qRT-PCR in liver tissues derived from YAC128 mice. E Protein levels for mHTT were analyzed by Western blotting with antibody MAB2166 in liver tissues derived from C6R mice. Representative blots and pooled quantification data with S.E.M. are shown, the blot matching panel C is shown in Fig. 5d. Statistical significance was determined by 1way-ANOVA (A) or two-tailed Student’s t-test (B-E), number of replicates is shown as insets. (TIFF 1130 kb) [file 40478_2018_518_MOESM5_ESM.tif]

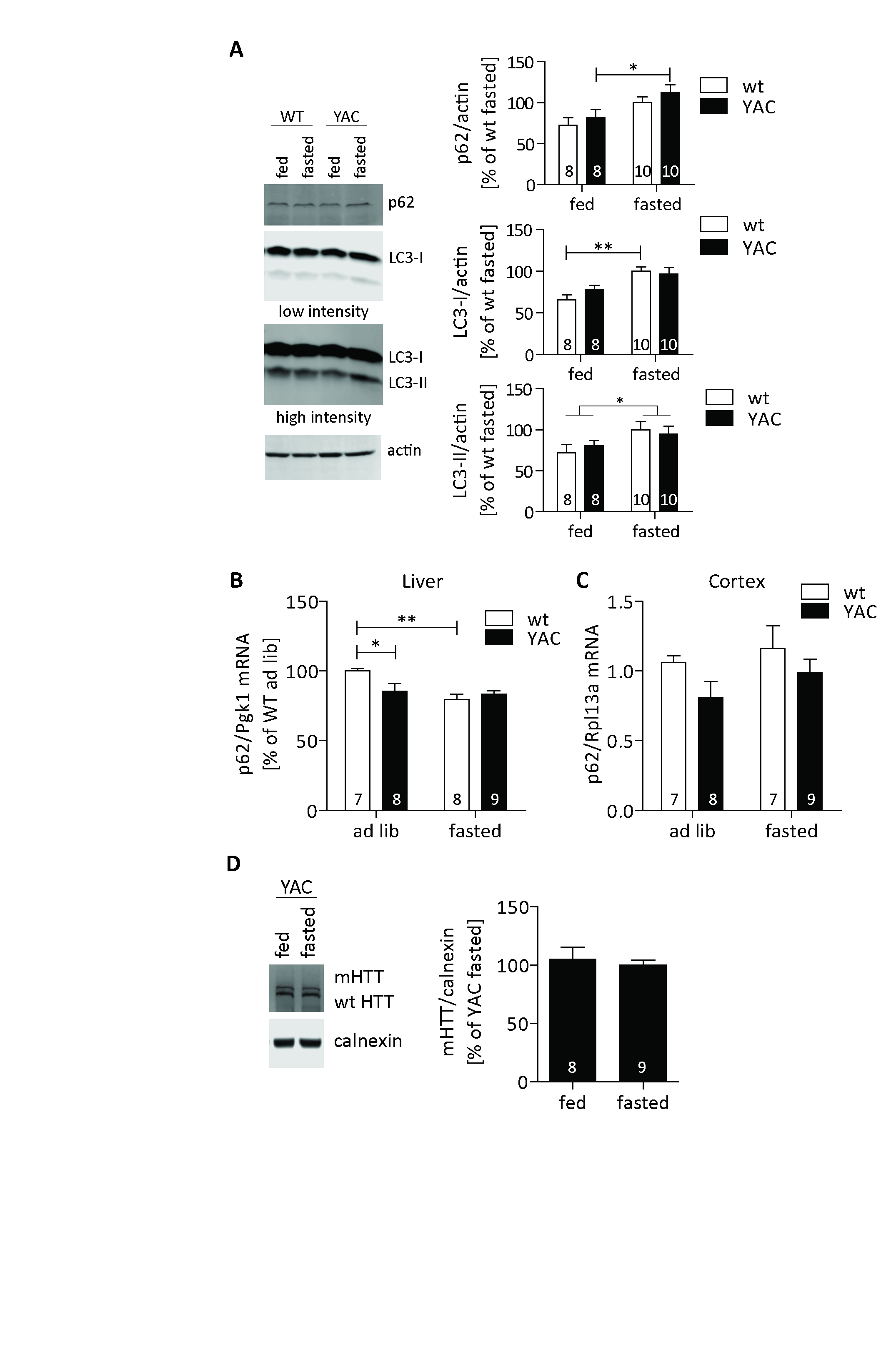

Supplement: Supplementary file 6 — Figure S6. 24h fasting increases autophagy but does not cause mHTT degradation in the brain. A + B 3 month old YAC128 and C6R mice as well as their wt littermates were subjected to a 24h fasting period, sacrificed immediately and cortical samples were compared to littermates with ad libitum access to food. A Protein levels of p62, LC3-I and LC3-II were analyzed by Western blotting in cortical tissues of wt and YAC128 mice. p62: 2way-ANOVA genotype p=0.2568, feeding p=0.0002, LC3-I: 2way-ANOVA genotype p=0.7914, feeding p<0.0001, LC3-II: 2way-ANOVA genotype p=0.5499, feeding p=0.0039. B Protein levels of mHTT were analyzed by Western blotting with antibody MAB2166 in cortical tissues of YAC128 mice. Representative blots and pooled quantification data with S.E.M. are shown, number of replicates is shown as insets. Statistical significance was determined by 2way-ANOVA with Bonferroni’s post-hoc correction for A, or two-tailed Student’s t-test for B. *: p<0.05, **: p<0.01, ***: p<0.001. (TIFF 1574 kb) [file 40478_2018_518_MOESM6_ESM.tif]

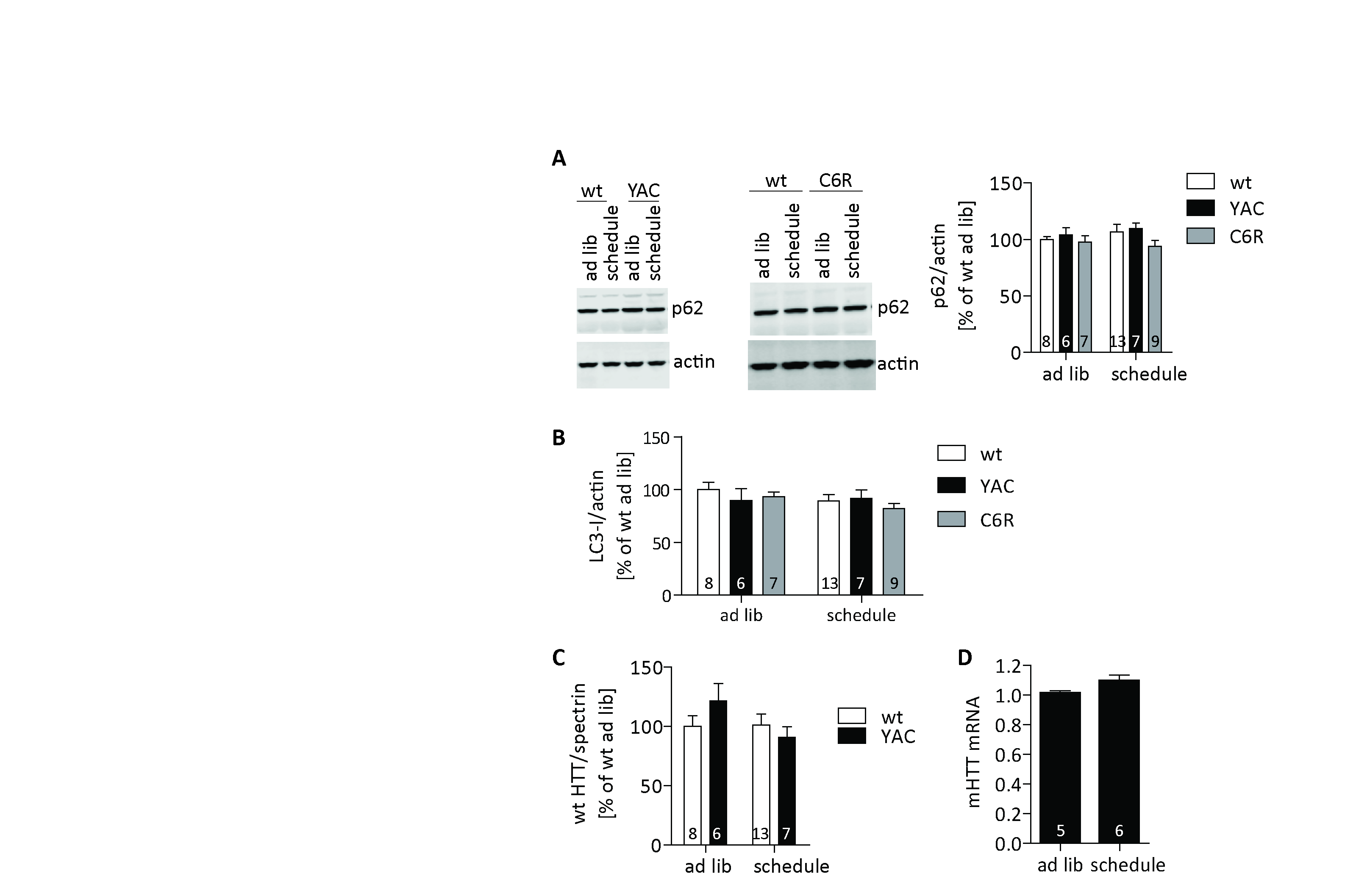

Supplement: Supplementary file 7 — Figure S7. Cortical p62, LC3-I and wt HTT protein levels as well as mHTT mRNA are not altered by scheduled feeding. A YAC128 and C6R mice as well as their wt littermates were subjected to one week of scheduled feeding and compared to littermates with ad libitum access to food. Protein levels of p62 were analyzed by Western blotting in cortical tissues. 2way-ANOVA, genotype p=0.2138, feeding p=0.5807. B YAC128 and C6R mice as well as their wt littermates were subjected to one week of scheduled feeding and compared to littermates with ad libitum access to food. Protein levels of LC3-I were analyzed by Western blotting in cortical tissues. 2way-ANOVA, genotype p=0.5798, feeding p=0.2548. C + D YAC128 mice and their wt littermates were subjected to one week of scheduled feeding and compared to littermates with ad libitum access to food. C Protein levels of wt HTT were analyzed by Western blotting with antibody MAB2166 in cortical tissues. 2way-ANOVA genotype p=0.6115, feeding p=0.1818. D mRNA levels for transgenic human mHTT were analyzed by qRT-PCR in cortical tissues derived from YAC128 mice. Representative blots and pooled quantification data with S.E.M. are shown, number of replicates is shown as insets. The blot corresponding to panel B is shown in Fig. 6a, the blot corresponding to panel C is shown in Fig. 6d. Statistical significance was determined by 2way ANOVA (A-C) or Student’s t-test (D). (TIFF 1304 kb) [file 40478_2018_518_MOESM7_ESM.tif]
